# Diversity roles of CHD1L in normal cell function and tumorigenesis

**DOI:** 10.1186/s40364-021-00269-w

**Published:** 2021-03-04

**Authors:** Xifeng Xiong, Xudong Lai, Aiguo Li, Zhihe Liu, Ningfang Ma

**Affiliations:** 1 Guangzhou Institute of Traumatic Surgery, Guangzhou Red Cross Hospital, Jinan University, Guangzhou, 510220 China; 2 Departement of infectious disease, Guangzhou Red Cross Hospital, Jinan University, Guangzhou, 510220 China; 3 Affiliated Cancer Hospital and Institute of Guangzhou Medical University, Guangzhou, 510095 China; 4 Department of Histology and Embryology, Guangzhou Medical University, Xinzao Town, Panyu District, Guangzhou, 511436 China

**Keywords:** CHD1L, ALC1, SNF2, Chromatin remodeling, Tumorigenesis

## Abstract

Chromodomain helicase/ATPase DNA binding protein 1-like gene (CHD1L) is a multifunctional protein participated in diverse cellular processes, including chromosome remodeling, cell differentiation and development. CHD1L is a regulator of chromosomal integrity maintenance, DNA repair and transcriptional regulation through its bindings to DNA. By regulating kinds of complex networks, CHD1L has been identified as a potent anti-apoptotic and pro-proliferative factor. CHD1L is also an oncoprotein since its overexpression leads to dysregulation of related downstream targets in various cancers. The latest advances in the functional molecular basis of CHD1L in normal cells will be described in this review. As the same time, we will describe the current understanding of CHD1L in terms of structure, characteristics, function and the molecular mechanisms underlying CHD1L in tumorigenesis. We inference that the role of CHD1L which involve in multiple cellular processes and oncogenesis is well worth further studying in basic biology and clinical relevance.

## Introduction

The *CHD1L* gene (*Chromodomain helicase/ATPase DNA binding protein 1-like gene*), also called the *ALC1* gene (*amplified in liver cancer 1*), locates on chromosome 1q21 region of human hepatoma cells and is cloned by Guan using the comparative genomic hybridization (CGH) technique [1–3]. Because CHD1L has a consistent helicase sequence motif found in helicase superfamily 2 proteins [1], it is classified as a sucrose non-fermentation 2 like (SNF2-like) subfamily of the SNF2 family [4–6]. Most SNF2-like proteins can utilize the energy, which is released from their DNA-dependent ATPase activity, to stabilize or interfere with protein-DNA interactions [7] and participate in a variety of nuclear activities, such as transcriptional inhibition or activation, DNA recombination and repair [8, 9]. Like most SNF-like proteins, CHD1L is a regulator of chromosome integrity, transcriptional regulation and DNA repair through its bindings to DNA. The functional diversity of CHD1L has always been related to the characteristics of helicases or chromatin remodeling enzymes that interact with PAR and catalyze the sliding of nucleosomes stimulated by PAR polymerase 1 (PARP1) [10, 11].

CHD1L plays an important role as a transcription and translation activator of its target genes [1, 6, 12–19]. CHD1L can promote cell proliferation, enhance cell migration and inhibit apoptosis by regulating various complex networks [6, 12, 13]. For example, CHD1L is a well-known activator of ARHGEF9, TCTP, SPOCK1 and NTKL [14–17] and can also lead to deregulation of p53, TCTP and Nur77 [1, 15, 18]. Importantly, CHD1L shows carcinogenicity in the process of malignant transformation. CHD1L overexpression in cancer cells is considered as a biomarker of short tumor-free survival time and poor prognosis [12, 20–28].

We will provide an overview of current understanding about CHD1L on structure, characteristics, function and the molecular mechanisms underlying CHD1L in cancer development in this review. As the same time, we will describe the latest development of CHD1L functions in normal cells. Finally, we will conclude that CHD1L is an attractively clinical target in the future molecular therapy of cancer.

### Molecular and structural features of CHD1L

#### SNF2 superfamily

CHD1L protein belongs to the SNF2 superfamily proteins, which was identified by Ma [1]. The SNF2 superfamily proteins include ATP-dependent chromatin remodeling enzymes and play key roles in the organization of genomic DNA in the natural chromatin state [7, 29]. The SNF2 superfamily proteins are further divided into ISWI (simulated switches), INO80 (inositol), CHD (chromosomal domain helicase DNA binding) and SWI/SNF (mating switch/non-sucrose fermentation) families [29]. In the mammalian, the ISWI family comprises SNF2H and SNF2L. The SWI/SNF family proteins contain brahma (BRM) and brahma-related gene 1 (BRG1). Based on the existence or nonexistence of additional domains, the CHD family contains three subfamilies: Chd1-Chd2, Chd3-Chd4, and Chd5-Chd9 [29]. The CHD family has the characteristics of two signature sequence motifs. One is the conserved SNF2_N domain at the N-terminal tandem chromodomain, and the other is the SNF2-like ATPase domain at the center, also known as the helicase superfamily c-terminal domain (HELICc) [9]. The SNF2_N domains (containing 280 amino acids (aa)) of CHD1L and CHD1 have 45% identity, while their HELICc domains (containing 107 amino acids) have 59% identity [1].

**Fig. 1:**
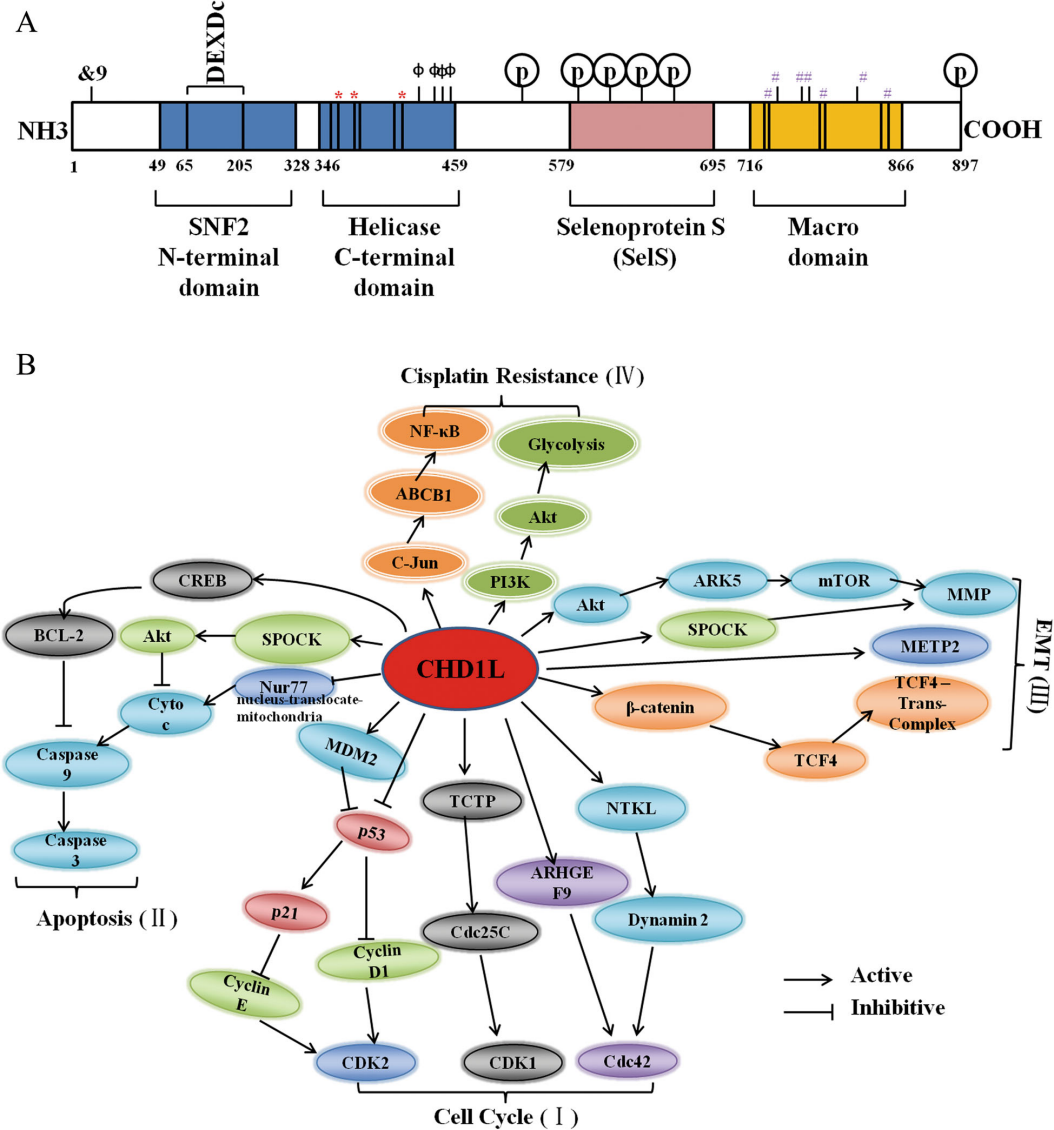
Structural representation, transcriptional effects and regulatory pathways of CDH1L. **a** Schematic representation of CHD1L protein. CHD1L encompasses four domains, SNF2 family N-terminal domain (49–328 aa, blue color) containing the ATP-binding region in N-terminal, helicase C-terminal domain (346–459 aa, blue color) containing nucleotide binding region, Selenoprotein S (SelS) (579–695 aa, pink color) consisting of several mammalian SelS sequences and a macro domain (716–866 aa, yellow color) containing ADP-ribose binding region in C-terminal. &9:Omega-N-methylarginine, no additional details recorded. DEXDc (65–205 aa):DEAD-like helicases superfamily. A diverse family of proteins involved in ATP-dependent RNA or DNA unwinding. This domain contains the ATP-binding region (74–78 aa, ATP binding site) and DEAH box (174–177 aa, putative Mg++ binding site). *: nucleotide binding region (371–374, 394–395, 421–423 aa). **#**: putative ADP-ribose binding sites (723–724, 741, 748, 750–751, 844, 846–848 aa). ɸ: ATP-binding sites (429, 450, 454, 457 aa). There are six putative phosphorylation sites: phospho-serine at 540, 607, 618, 628, 636 and 891 amino acids, respectively. **b** The transcriptional effects and regulatory pathways of CDH1L. (I) CHD1L accelerate cell cycle transition, which directly binds to the promoter regions of MDM2, P53, TCTP, ARHGEF9 and NTKL. (II) CHD1L promotes cell apoptosis death through activing caspase pathway, which enhances the phosphorylation of CREB, increases SPOCK expression and inhibits nuclear to mitochondrial translocation of Nur77. (III) CHD1L activate the transcription of Akt, METP2, TCF4 genes, leading to EMT (include invasion and metastasis). (IV) CHD1L regulates NF-κB and glycolysis pathways to facilitate cisplatin resistance

*CHD1L* is mapped to chromosome 1q21 [30]. The full-length messenger RNA of CHD1L (NM_004284.6) contains 3036 base pairs with a presumptive open reading frame encoding an 897 aa protein [1]. As shown in Fig. 1a, CHD1L mainly contains four domains: a conserved SNF2_N domains, a helicase superfamily domains (HELICc), selenoprotein S (SelS) and a Macro domains [1]. The upstream of *CHD1L* gene is flavin containing monooxygenase 5 (*FMO5*) gene, while the downstream of which have a long intergenic non-coding RNA 624 (LINC00624) and a prostaglandin r*eductase pseudo* gene (LOC100130018) [30]. In addition, human CHD1L has a Selenoprotein S (SelS) region (579–695 aa), which is a plasma membrane protein that exists in many cell types and tissues [31]. Schematic representation of human CHD1L protein is shown in Fig. 1a.

#### Characteristics and experssion pattern

**Fig. 2:**
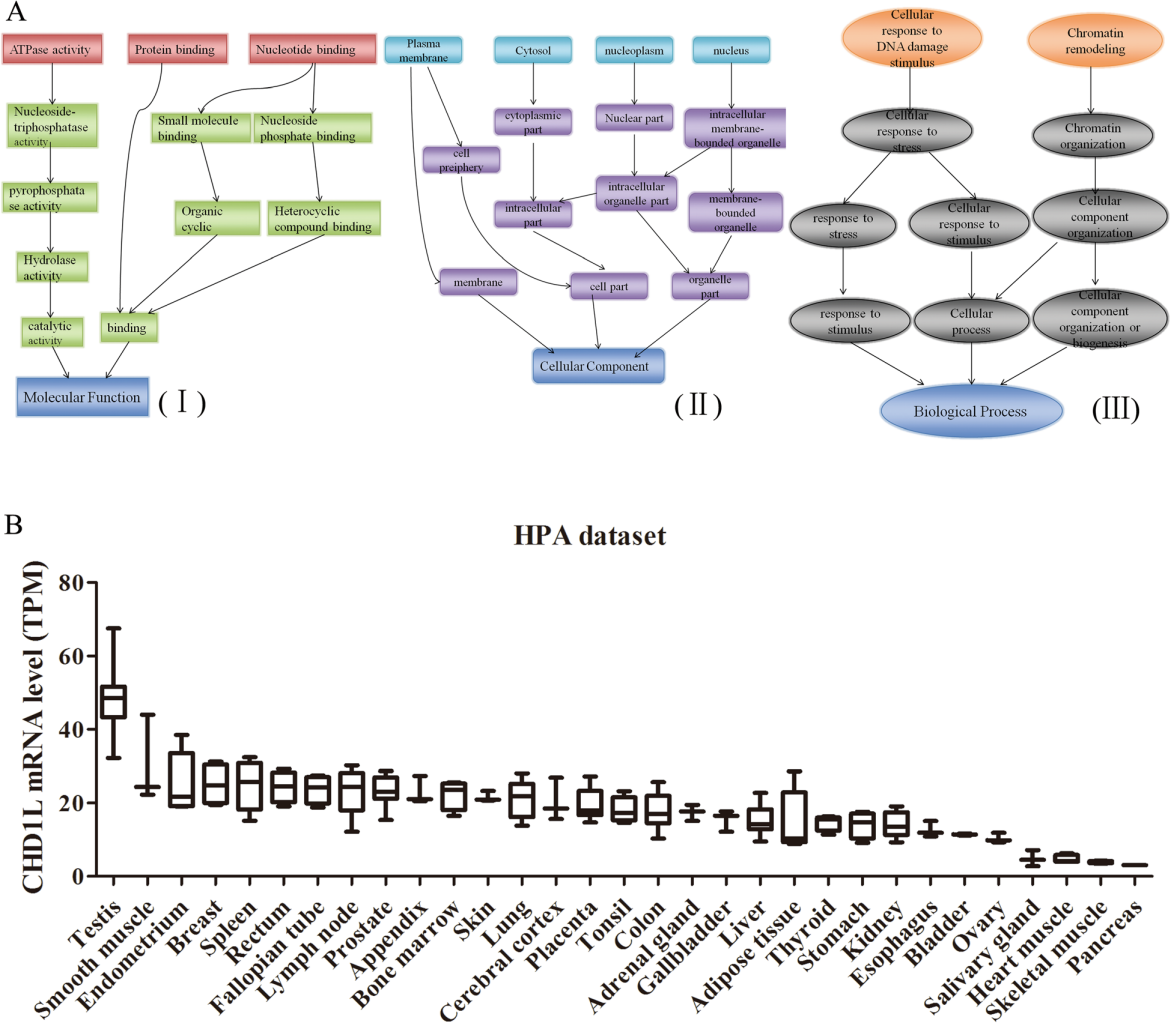
Characteristics and experssion pattern of CHD1L. **a** Conserved gene CHD1L in eukaryota (HomoloGene ID:11590) involves in molecular function (I), cellular component (II), biological process (III)., cellular component, biological process. **b** CHD1L mRNA expression overview of tissue category from HPA dataset. TPM: Transcript Per Million

CHD1L is a highly conserved gene among species in nature (https://www.ncbi.nlm.nih.gov/homologene/?term=11590). Human CHD1L is more than 87, 73, and 66% similar to mammals, the less-evolved vertebrate chicken and zebrafish, respectively (Table 1). As conserved gene in eukaryota (HomoloGene ID: 11590), CHD1L is involved in molecular function, cellular component, biological process (Fig. 2a, Table 2) (http://www.informatics.jax.org/homology/GOGraph/11590#Annotations). Since CHD1L is expressed in many species including invertebrates, it appears that CHD1L exists in the common ancestor of vertebrates. In human, CHD1L can be found in various tissues and exhibit different expression patterns in space and time [22] (Fig. 2b). For example, in the male reproductive system (testis), CHD1L is less expressed in mature cells than progenitor cells [32, 33].

**Table 1:** conserved gene CHD1L homology in Eukaryota (HomoloGene:11590) [https://www.ncbi.nlm.nih.gov/homologene/?term=11590]

HomoloGene:11590. Gene conserved in Eukaryota						
Species	Symbol	Genetic location	Protein Acc.	Protein length	Identity(%)^a^	
					protein	DNA
Human (H.sapiens)	CHD1L	Chr1 q21.1	NP_004275.4	897 aa		
Mouse (M.musculus)	Chd1l	Chr3 42.17 cM	NP_080815.1	900 aa	87.6	85.3
Rat (R.norvegicus)	Chd1l	Chr2 q34	XP_006233080.1	926 aa	88.2	86.1
Chimpanzee (P.troglodytes)	CHD1L	Chr1	XP_001158033.1	896 aa	99.0	99.1
Cattle (B.taurus)	CHD1L	Chr3	NP_001032909.1	897 aa	91.6	90.3
Dog (C.lupus)	CHD1L	Chr17	XP_005630931.1	918 aa	88.0	88.5
Chicken (G.gallus)	CHD1L	Chr1	XP_004938249.1	900 aa	73.4	72.6
Zebrafish (D.rerio)	chd1l	Chr6	NP_956607.1	1026 aa	66.5	64.1

^a^VS Human (H.sapiens)

**Table 2:** Function of CHD1L in HomoloGene:11590 (http://www.informatics.jax.org/homology/GOGraph/11590#Annotations)

	Category	Classification term	Gene Ontology ID^a^	Reference
Full set of experimental annotations for Human-Mouse-Rat CHD1L genes in HomoloGene:11590	Molecular Function	ATPase activity	GO:0016887	[11]
		nucleotide binding	GO:0000166	
		protein binding	GO:0005515	
	Cellular Component	plasma membrane	GO:0005886	^b^
		cytosol	GO:0005829	
		nucleoplasm	GO:0005654	
		nucleus	GO:0005634	[11]
	Biological Process	cellular response to DNA damage stimulus	GO:0006974	[11]
		chromatin remodeling	GO:0006338	

^a^http://www.informatics.jax.org/vocab/gene_ontology/

^b^http://www.informatics.jax.org/go/reference/J:164563

#### Biological function

Studies have described five spliced variants of the CHD1L protein, and even found six spliced transcribed variants of the *CHD1L* gene [30]. Interestingly, CHD1L is a conserved protein with four conserved protein domains, namely SNF2_N domain, HELICc, SelS and Macro domain (Fig. 1a).

SNF2_N domain is an important part of many proteins that involved in a variety of cell biological processes including DNA repair, chromatin unwinding, DNA recombination, and transcription regulation, but also is composed of some proteins with little functional information [9, 34].

HELICc is a component of multiple helicases and helicase-related proteins and DEAD-, DEXDc-, DEAH-box associated proteins [35, 36], hepatitis C virus NS3, ski2p and yeast initiation factor 4A [37]. The HELICc is not an autonomous folding unit, but is an essential part of the helicases that utilize the energy from nucleoside triphosphate hydrolysis to provide fuel for their translocation along DNA and unwinding double-stranded DNA in the process [36, 38]. The HELICc also contains DEAD-like helicase superfamily (ATP-binding) region which participates in ATP-dependent DNA or RNA unwinding.

SelS family contains several mammalian SelS sequences. SelS is a disordered protein which has a seleno sulfide bond (between Cys-174 and Sec-188) and a redox potential (− 234 mV) [31]. SelS is an efficient reductase that can catalyze the reduction of hydrogen peroxide [39]. SelS also has the ability to resist hydrogen peroxide inactivation and may have an evolutionary advantage compared to cysteine-containing peroxidases [40].

Macro domain is a high-affinity ADP-ribose binding module, which exists in various proteins in the form of independent domains or complexed with other domains (such as poly ADP-ribose polymerase (PARPs) and histone macroH2A). Poly ADP-ribose can be recognized as a ligand by some macro domains. Initially, the macro domain was identified as performing ADP-ribose-1″-monophosphate (Appr-1″-p) processing activity. Besides, the macro domain also play important roles in different ADP-ribose pathways, including DNA transcription, chromatin biology, DNA repair and long-term memory formation [34].

### Roles of CHD1L in chromatin remodeling

The CHD1L protein includes highly conserved helicase motifs that exist in other SNF2 family members such as CHD1, ISWI and Snf2 [41]. However, unlike CHD1, CHD1L contains a macro domain that can recognize poly ADP-ribose (PAR), but not a chromo domain that recognizes methylated histone tails [11, 42]. PAR is produced by PAR polymerase (PARP), and single-stranded DNA breaks (SSB) and gaps can activate PARP [43, 44]. The occurrence of SSB is during base-excision repair (BER) [45] and can eliminate base damage, including alkylation and oxidation [45, 46]. The formation of PAR on chromatin protein near SSB promotes the recruitment of CHD1L and other BER factors to damaged bases [47]. CHD1L promotes BER by spreading chromatin at the sites of DNA damage [48]. PARP1 stimulates the chromatin-repositioning activity of CHD1L [10, 49]. CHD1L contributes to PARP-dependent BER without affecting the recruitment of DNA damage by XRCC1 or Polβ [48]. Chromatin relaxation is one of the earliest responses of cells to DNA damage, PARP1 triggers a conformational change that activates CHD1L to drive chromatin relaxation [50–52]. The co-operation between CHD1L and PARP is also related to the nucleotide excision repair of UV-induced DNA damage [53]. Another important role of CHD1L is involved in the transcriptional control for DNA damage responses, which supports the fact that CHD1L interacts with Tripartite Motifcontaining 33, a multifunctional protein involved in transcriptional regulation [54]. In addition, CHD1L promotes cell tolerance by slowing the site of DNA damage (as a chromatin remodeler) induced by replication forks of camptothecin, a topoisomerase I toxin that produces single strand breaks and causes the breakdown of replication forks [55]. CHD1L-dependent nucleosome remodeling is required for the efficient handover between PARP1/2, DNA glycosylases, and APEX1 downstream of lesion excision. Loss of ALC1 confers methyl-methanesulfonate, PARP inhibitors and formyl-dU sensitivity, which is synthetic lethal with homologous recombination deficiency (HRD) [56]. Therefore, CHD1L not only has a PAR-dependent chromatin remodeling activity, but also promotes DNA repair reactions within the chromatin range.

### The critical role of CHD1L in development and differentiation

The developmental processes of the mammalian embryo are routinely analyzed according to their potential genetic components. Among many genes which are characterized by their roles in the early embryonic development, especially in pre-implantation [57], CHD1L is a key developmental regulator and is necessary for the early stages of development [58–60]. Inhibiting the production of CHD1L can arrest embryo at the pre-blastocyst stage in mice embryonic stem (ES) cells by microinjecting antisense morpholinone [58]. CHD1L regulates stem cell pluripotency through interplaying with PARP1 during early developmental stage [59]. However, CHD1L is not necessary for the survival, pluripotency and differentiation of cultured ES cells [58]. CHD1L is essential for embryonic events that are distinct from events in ES cells [58].

CHD1L is developmentally regulated and expressed in human fetuses, with the highest expression of CHD1L in the brain, followed by the kidney, then muscle, liver, thymus, lung, heart, and spleen [61]. CHD1L is indispensable for the development of human embryonic neuroepithelium. CHD1L overexpression in hESCs promoted neuroepithelial differentiation in both self-renewal and directional differentiation conditions. CHD1L knockdown impaired hESC differentiation into neuroepithelium. CHD1L overexpression dramatically upregulated PAX6 expression which is a key regulatory gene in eye and brain development. Interestingly, CHD1L highly expressed in cells of the ventricular (germinal) zone of E14 mouse embryos, and it colocalized with PAX6-positive cells [60]. The expression of CHD1L is higher in the kidney of fetal than in adult (4:1). This fact means that CHD1L is particularly important in kidney development [61]. CHD1L also plays a key role in congenital anomalies of the kidneys and the urinary tract (CAKUT). Three different heterozygous missense variants of CHD1L (variant Gly700Arg, variant Ile765Met and variant Ile827Val) were revealed by sequencing the entire coding region of the *CHD1L* gene in 61 CAKUT patients and exons 18, 19 and 21 in 24 CAKUT patients. The interaction between all three CHD1L variants and PARP1 decreased compared with the wild-type CHD1L. Therefore, chromatin remodeling and ATPase activities of CHD1L are low in CAKUT [61].

In adult, CHD1L expression is the highest in the testis, and it is mainly localized in undifferentiated spermatogonia, suggesting the role of CHD1L in spermatogenesis [32, 61]. Spermatogenic stem cells (SSCS) are adult stem cells, which are parthenogenetic cells based on spermatogenesis and male fertility [33]. CHD1L is a novel and intrinsic regulator of SSCs self-renewal and survival, which is at least partially mediated by the GDNF signaling pathway [32].

### Molecular mechanism of CHD1Lin tumorigenesis

Multiple studies have shown that CHD1L plays important roles in cell proliferation, cell metastasis, cell apoptosis, cell cycle transition and drug resistance through a variety of mechanisms. CHD1L can promote G1/S transition and DNA synthesis by upregulating cyclins, CDK2, 4 and downregulating P27, Rb and p53 in transgenic mouse models [62]. In the development of HCC, CHD1L has been shown to participate in many ways, such as CHD1L-ARHGEF9-CDC42-EMT axis [14], CHD1L-TCTP-CDC25C-CDK1 pathway [15], CHD1L-SPOCK1-Akt signaling pathway [16]. In breast cancer, CHD1L promoted cell metastasis and invasion through the PI3K/AKT/ARK5/mTOR/MMP pathway [4]. CHD1L might promote cell motility and cell cycle progression through the MDM2/p53 pathway [63]. CHD1L could induce G1/S transition by the dysregulation of p53-cyclinE-CDK2 pathway in glioma [64]. CHD1L influenced cell proliferation by activating the Wnt/β-catenin/TCF pathway in pancreatic cancer [65]. The dysregulation of p53-cyclin D1-CDK2 pathway might be related to CHD1L-induced G1/S transition, while CHD1L might drive EMT and MET and cause metastasis of cholangiocarcinoma cells [20]. In terms of drug resistance, the upregulation of CHD1L could promote cisplatin resistance of NSCLC cells through c-Jun/ABCB1/NF-κB axis [66]. However, the downregulation of CHD1L enhanced cisplatin cytotoxicity of esophageal squamous cell carcinoma cells by inhibiting the glycolysis of PI3K/AKT pathway [67]. Taken together, inappropriate expression of CHD1L target genes and deregulation of CHD1L system may link CHD1L to tumorigenesis by several mechanisms (Fig. 1b).

### Transcriptional effects of CHD1L on target genes

#### ARHGEF9

ARHGEF9, also called Collybistin, is one of the guanine nucleotide exchange factor (GEF) superfamily, which catalyzes GDP-GTP exchange in small GTPases of Rho family [68, 69]. An important molecular mechanism of cancer metastasis is the activation of the Rho family of small GTPases, which leads to the rearrangement of the actin cytoskeleton and regulates cadherin-dependent cell-to-cell contacts [70–72]. Most mammalian GEFs targeting Rho GTPases can accelerate cancer cell invasion by enhancing GTP loading on Rho proteins [73]. ARHGEF9 can encode a special guanine nucleotide exchange factor (GEF) for the Rho small GTPase Cdc42 [74]. ARHGEF9 is a target gene of the transcriptional regulator CHD1L [14]. CHD1L can upregulate transcription of ARHGEF9, which then increases Cdc42 activity, causing EMT and finally invasion and metastasis of HCC. CHD1L-ARHGEF9-Cdc42-EMT might be a novel pathway to participate in the progression and metastasis of HCC [14].

#### SPOCK1

Sparc/osteoectin, cwcv and kazal-like domain proteoglycan 1 (SPOCK1) is a secreted protein and can encode a Ca2 + −binding matricellular glycoprotein, which belongs to acidic and rich in cysteine (SPARC) family [75]. The SPARC family plays a role in cell migration, cell proliferation and cell apoptosis of certain types of cancer [76]. As a member of the family, SPOCK1 also plays a key role in proliferation, adhesion and migration of cancer cells [77–79]. CHD1L can activate transcription of SPOCK1 by binding to the 5’upstream region of SPOCK1, and promote anti-apoptotic effect by activating the AKT pathway and inhibiting the cytochrome c/caspase-9/caspase-3 pathway [16].

#### TCTP

Translationally controlled tumor protein (TCTP) expresses in almost all mammalian tissues as a housekeeping gene. TCTP is a pro-survival factor by inhibiting apoptosis and promoting cell cycle as a tubulin-binding protein [80]. CHD1L protein can bind directly to the 5′ upstream region (nt − 733/− 1027) of TCTP and activate its transcription [15]. Then, TCTP could promote the ubiquitin proteasome degradation of CDC25C, and downregulate CDK1 activity by inhibiting the dephosphorylation of CDK1 at Tyr15 and lead to a faster mitotic exit during mitotis [15]. The CHD1L-TCTP-CDC25C-CDK1 pathway could cause malignant transformation of hepatocytes, and its phenotype accelerated the mitotic process and produced aneuploidy [15].

#### MDM2

MDM2 is characterized by dynamic negative regulation of the tumor suppressor p53 [81], which can promote cell cycle transition in p53-dependent [82] and p53-independent [83] manner. Increased levels of MDM2 could promote the ubiquitination and degradation of E-cadherin, which in turn drove cancer cell invasion [84]. CHD1L might facilitate the progress of breast cancer cells via the MDM2/p53 signaling pathway [63].

#### NTKL

N-terminal kinase like protein gene (NTKL) locates on 11q13 and encodes a 808 amino acid protein [85], which exhibited in golgi apparatus, centrosomes, cytoplasm and nucleus of subcellular localizations [86]. Golgi NTKL was reported to interact with Cop1 vesicles and regulate golgi morphology [87], while centrosome NTKL played an important role in cell division [88]. NTKL was frequently upregulated by CHD1L in primary HCC cases and exhibited a strong oncogenic ability [17].

#### ABCB1

The ATP-Binding Cassette Sub-Family B Member 1 (ABCB1), also called the plasma membrane glycoprotein (P-glycoprotein), locates on the chromosome 7q21.12, which encodes a 170 KD trans-membrane glycoprotein and belongs the ATP-binding cassette (ABC) transporters family [89]. The ABC transporter family has a transport effect on chemotherapeutic agents, which causes the occurrence and development of multidrug resistance (MDR). ABCB1 is the most important resistance-inducing protein. ABCB1 was a potential downstream target gene of CHD1L in NSCLC cells [66]. The up-regulation of ABCB1 by CHD1L depended on the transcription of c-Jun. ABCB1 knockdown coupled with CHD1L ectopic expression enhanced the effect of cisplatin on apoptosis of NSCLC cells [66].

#### β-Catenin

β-catenin is a typical influencer of Wnt signaling pathway and a component of cell-cell adhesion complex, which participates in cell proliferation, metastasis, differentiation and tumorigenesis [90, 91]. When the Wnt signaling pathway is activated, β-catenin accumulates in the cytoplasm and then enters the nucleus to form a complex with the nuclear transcription factor Tcf/Lef. Then, β-catenin can activate a series of downstream target genes, which participate in the transcription process in different ways [92]. CHD1L overexpression significantly increased β-catenin expression in pancreatic cancer [65]. However, elevated β-catenin expression exhibited in the CHD1L-KD group in glioma [64]. Therefore, β-catenin may play different roles in different tumors as a target gene of CHD1L.

#### p53

p53 protein is essential for effective suppression of human tumors [93]. Overexpression of CHD1L inhibits the expression of p53 in HCC [1], breast cancer [63] and glioma [64], makes p53 lose its anti-cancer effect. p53 protein can upregulate p21 expression, and the latter acts as a Cdk2 inhibitor, inactivating cyclinE-CDk2 complex to control the S phase entry [62, 94, 95]. CHD1L overexpression could promote cell proliferation by downregulating the p53-p21-cyclinE-Cdk2 pathway in HCC [1]. However, the loss of CHD1L resulted in increased expression of p53 and p21, while decreased expression of cyclinE and Cdk2 in glioma [64].

#### Nur77

Nur77, also named NR4A1, is a unique transcriptional factor and belongs to the orphan nuclear receptor superfamily [96, 97]. Nur77 is a key member of the p53-independent apoptotic pathway, which directly targets Bcl-2 and induces the latter to adopt a pro-apoptotic conformation, thereby triggering the release of cytochrome c and the activation of caspase-9 and caspase-3 [98–100]. CHD1L can inhibit the nuclear translocation of Nur77 to mitochondria. Macro domain of CHD1L acts to interact with Nur77, while the CHD1L mutants lacking residues 600–897 cannot interact with Nur77 and prevents Nur77-mediated apoptosis of hepatocellular carcinoma [18].

#### hMLH1

Human mutL homolog 1 gene (*hMLH1*) is located on the chromosome 3p21.3–23, which encodes a protein of 756 amino acids. hMLH1 is an important member of DNA mismatch repair (MMR) protein family. MMR is a common DNA repair process which helps maintain the stability and integrity of genetic substances [101]. It has been observed that hMLH1 protein interacts with mismatched genes and DNA repair enzymes, and enhances DNA repair [102]. Downregulation of hMLH1 could lead to the loss of DNA MMR repair function and promote tumor progress [102]. CHD1L can inhibit hMLH1 expression in cholangiocarcinoma cells, which was related to the malignant progression and prognosis of cholangiocarcinoma [103].

### CHD1L related disease

#### The homology and difference between CHD1L and CHD1

**Fig. 3:**
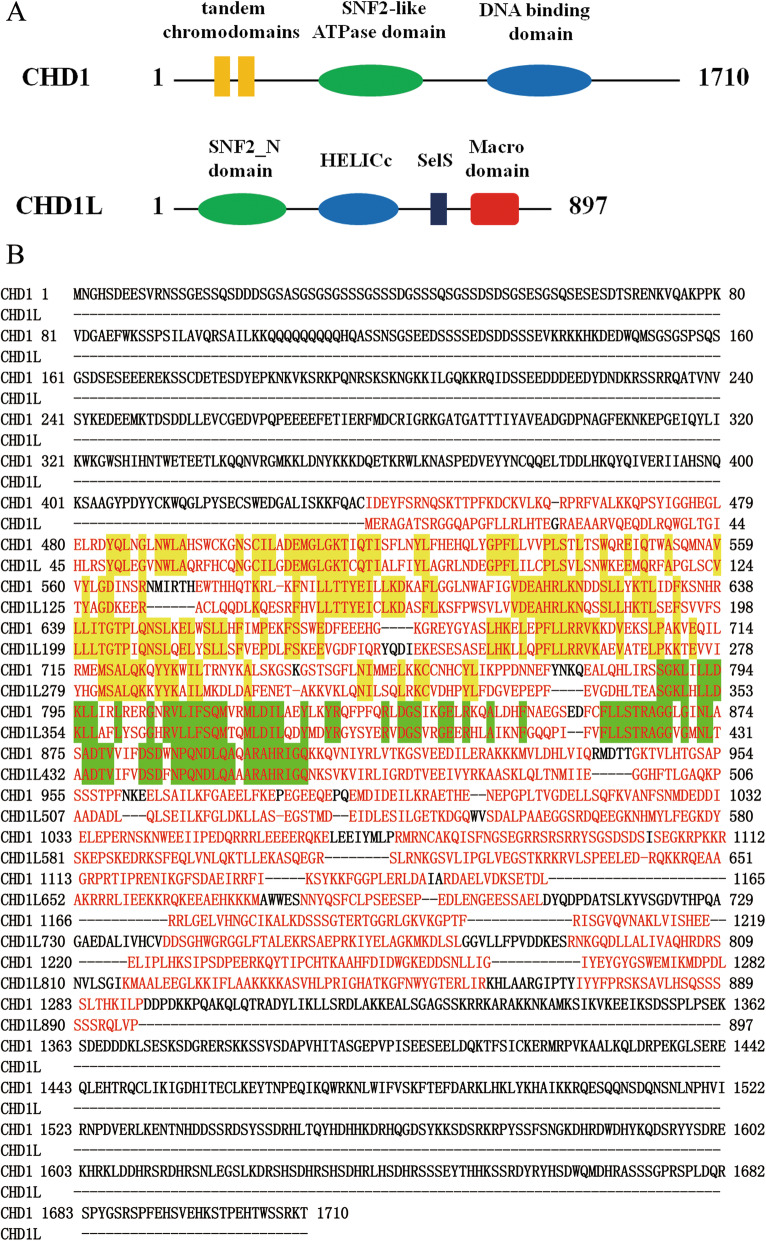
The homology and difference between CHD1L and CHD1. **a** Structural diagram of CHD1L and CHD1. CHD1 contains two N-terminal tandem chromodomains (yellow rectangles), a central SNF2-like ATPase domain (green oval), and a C-terminal DNA binding domain (blue oval). CHD1L contains a SNF2_N domain (green oval), HELICc (blue oval), Sels (black rectangle) and a Macro domain (red rectangle). **b** Multiple Alignment Results of CHD1L and CHD1 Protein Sequence. The sequence homology between the SNF2_N domain of CHD1L (280 aa) and SNF2-like ATPase domain of CHD1 have 45% identity (126/280, yellow indicator). The sequence homology between the HELICc domain of CHD1L (107 aa) and DNA binding domain of CHD1 have 59% identity (63/107, green indicator)

CHD1 locates in 5q15 and encodes a protein composed of 1710 amino acids (hCHD1: NP_001261), belongs to the Chd family proteins that play an important role in transcriptional regulation and developmental processes [29]. CHD1 is characterized by two N-terminal tandem chromodomains (yellow rectangles), a central SNF2-like ATPase domain (green oval), and a C-terminal DNA binding domain (blue oval) (Fig. 3a) [104]. Chromodomains are modules implicated in the recognition of lysine-methylated histone tails and nucleic acids. Lysine methylation at a specific site on the histone H3 tails is associated with transcriptional regulation. Methylation at H3 K4, H3 K36 and H3 K79 is linked to transcriptional activity, whereas methylation at H3 K9, H3 K27 and H4 K20 serves as a marker for epigenetic silencing [105]. The SNF2-like ATPase domain defines the ATP-dependent chromatin remodeling proteins. The Chd1 DNA-binding domain is consist of a SANT and SLIDE domain, which is required for efficient nucleosome sliding and believed to be essential for sensing the length of DNA flanking the nucleosome core [106].

Sequence homology analysis showed that CHD1L (initially called ALC1, hCHD1L: NM_004284.6) contains a conserved SNF2_N domain (green oval), a helicase superfamily c-terminal domain (HELICc) (blue oval), Sels domain (black rectangle) and a Macro domain (red rectangle) in human by Ma et al. (Fig. 3a) [1]. The SNF2_N domain is composed of 280 amino acids, and the sequence homology between the SNF2_N domain of CHD1L and SNF2-like ATPase domain of CHD1 have 45% identity (126/280, yellow indicator). The HELICc domain is composed of 107 amino acids, and the sequence homology between the HELICc domain of CHD1L and DNA binding domain of CHD1 have 59% identity (63/107, green indicator) (Fig. 3b). Therefore, the name of CHD1L was given.

The difference structures between CHD1L and CHD1 are that CHD1 has two tandem chromodomians that affect DNA functions, including transcription, replication, recombination and repair, while CHD1L uses the Marco domain to perform biological functions by binding PAR. The Macro domain proteins also recognize poly-ADP-ribose as a ligand, and the ADP-ribosylation of proteins is an important posttranslational modification that occurs in a variety of biological processes, including DNA repair, transcription, chromatin biology and long-term memory formation [42].

Studies have shown that CHD1 plays an important role in embryonic stem cell differentiation, hematopoietic stem cells emergence and tumorigenesis. Full length CHD1 is required in embryonic stem cell differentiation, and loss of the serine-rich region (SRR) renders CHD1 unable to support normal differentiation of ESCs into the three germ layers [107]. Endothelial-specific deletion of CHD1 leads to loss of definitive hematopoietic progenitors, anemia, and lethality by embryonic day (E)15.5 [108]. CHD1 is the 5q21 tumor suppressor gene, and inactivation of CHD1 abolishes recruitment of androgen receptor (AR) to result in downregulation of AR-responsive genes (eg. FOXO1, NKX3–1 and PPARγ) in prostate cancer [109]. Recurrent deletion of CHD1 is a driver of prostate cancer cell invasiveness [110]. CHD1 drives immune suppression in PTEN-deficient prostate cancer [111]. a novel CHD1-RUNX1 fusion collaborated with FLT3-ITD mutation in the development of acute myeloid leukemia [112]. However, CHD1L is an oncoprotein, which is overexpression in various cancers.

#### Abnormal expression and pan-cancer analysis of CHD1L

**Fig. 4:**
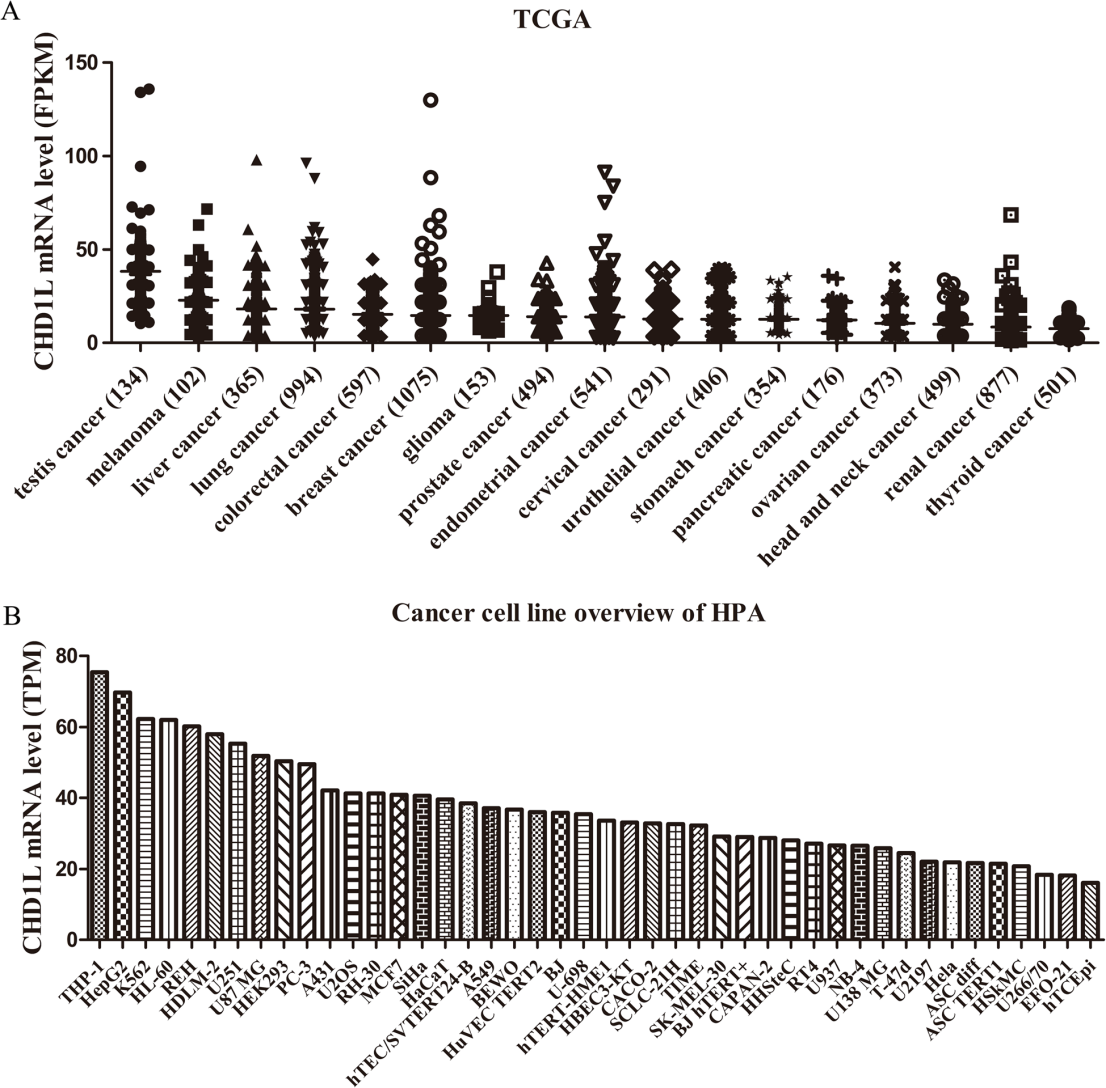
CHD1L mRNA expression overview of cancer category and cell lines from TCGA (**a**) and HPA (**b**) dataset. FPKM: Fragments Per Kilobase of exon per Million. TPM:Transcript Per Million

**Fig. 5:**
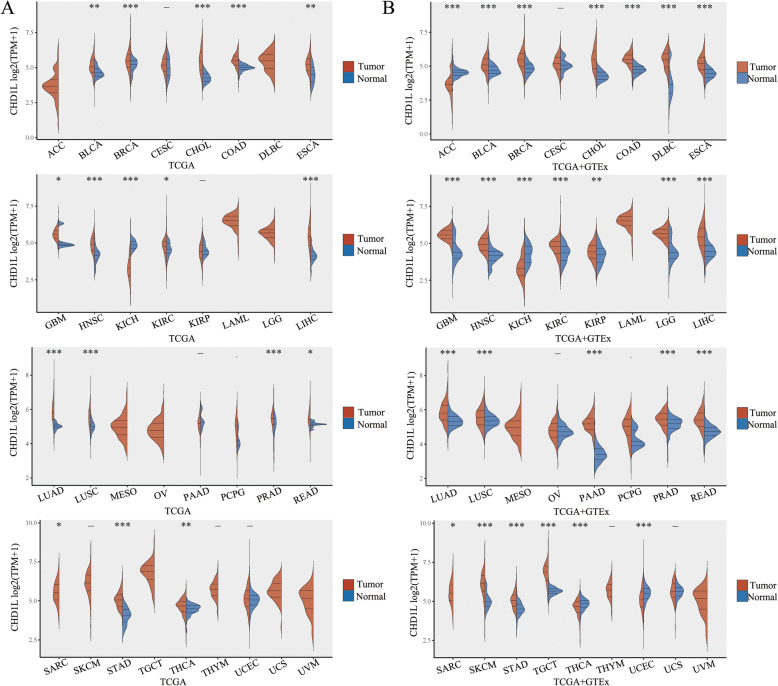
Pan-cancer analysis of CHD1L expression in TCGA dataset (**a**) and the TCGA combined GTEx dataset (**b**)

Several studies have found that CHD1L has a strong carcinogenic ability, including promoting tumor cell proliferation, invasion, migration, metastasis and inhibiting tumor cell apoptosis [1, 6, 12, 13]. In mESCs, overexpression of CHD1L increases tumor susceptibility [58]. It is documented that CHD1L is deregulation in various neoplastic diseases. CHD1L is an important oncoprotein that is overexpressed in various malignant tumors, including HCC [1, 14–18, 21], ovarian cancer [23], gastric cancer [22], colorectal carcinoma [24], bladder cancer [25], breast cancer [4, 26, 63], nasopharyngeal carcinoma [27], glioma [64], NSCLC [12, 66], myeloma [6], pancreatic cancer [65], esophageal carcinoma [28, 67] and cholangiocarcinoma [20, 103] (Fig. 4 and Table 3). Further, pan-cancer analysis of CHD1L expression was determined in The Cancer Genome Atlas (TCGA) dataset (https://tcga-data.nci.nih.gov/tcga/) and The Genotype-Tissue Expression (GTEx) project (https://gtexportal.org/). In TCGA dataset, the expression of CHD1L in BLCA, BACA, CHOL, COAD, ESCA, GBM, HNSC, KIRC, LIHC, LUAD, LUSC, PRAD, READ, SARC, STAD and THCA was higher than the normal tissues, while in KICH was lower than normal tissues, and the differences are statistically significant (Fig. 5a). In the TCGA combined GTEx database, CHD1L is expressed in BLCA, BACA, CHOL, COAD, DLBC, ESCA, GBM, HNSC, KIRC, KIRP, LGG, LIHC, LUAD, LUSC, PAAD, PRAD, READ, SARC, SKCM, STAD, TGCT, THCA and UCEC are all higher than normal tissues, while in ACC, KICH is lower than normal tissues, and the difference is statistically significant (Fig. 5b).

**Table 3:** Summary of the current literature on CHD1L deregulation in solid cancers

Cancer	Deregulation	Downstream targets	Phenotypic effect	clinical impact	References
HCC	CHD1L↑	P53↓, p21↓, cyclinE↑, CDK2↑	G1/S phase transition↑, apoptosis↓, proliferation↑		[1]
	CHD1L↑	Nur77↓	apoptosis↓		[18]
	CHD1L↑	ARHGEF9↑, Cdc42↑	migration↑, invasion↑, metastasis↑		[14]
	CHD1L↑	TCTP↑, Cdc25c↓, Cdk1↓	Mitotic progression↑, aneuploidy↑		[15]
	CHD1L↑	SPOCK1↑, Akt↑	apoptosis↓, invasion↑, metastasis↑		[16]
	CHD1L↑	NTKL↑	cell growth↑, colony formation↑, G1/S transition↑		[17]
	CHD1L↑	NA		Poor Prognosis	[21]
Ovarian carcinoma	CHD1L↑	NA	metastasis↑	Prognostic biomarker	[23]
	CHD1L↑	METAP2↑	invasion↑, metastasis↑		[19]
Gastric cancer	CHD1L↑	NA		Prognostic biomarker	[22]
Colorectal carcinoma	CHD1L↑	NA	G1/S phase transition↑, apoptosis↓	Prognostic biomarker	[24]
Bladder cancer	CHD1L↑	NA		Prognostic biomarker	[25]
Breast cancer	CHD1L↑	NA		Prognostic biomarker	[27]
	CHD1L↑	MDM2↑, p53↓	Cell cycle↑, cell motility↑		[63]
	CHD1L↑	PI3K↑, Akt↑, ARK5↑, mTOR↑, MMP2↑, MMP9↑	chemotaxis↑, invasion↑, lung colonization↑		[4]
Nasopharyageal carcinoma	CHD1L↑	NA		Prognostic biomarker	[27]
Glioma	CHD1L↑	PCNA↑, β-catenin ↓, cyclinD1↑, p53↓, p21↓, cyclinE↑, Cdk2↑, c-capase3↓, Bcl2↑	G1/S phase transition↑, proliferation↑, apoptosis↓, migration↑, invasion↑		[64]
NSCLC	CHD1L↑	NA		Prognostic biomarker	[12]
	CHD1L↑	ABCB1↑, c-Jun↑, NF-kB↑	Cisplatin resistance		[66]
Myeloma	CHD1L↑	c-capase9↓, capase3↓	Anti-apoptosis, cell adhesion-mediated drug resistance↑		[6]
Pancreatic cancer	CHD1L↑	β-catenin↑	Cell proliferation↑	Poor prognosis	[65]
Esophageal carcinoma	CHD1L↑	NA	proliferation↑, apoptosis↓, metastasis↑, invasion↑	Poor prognosis	[28]
	CHD1L↑	PI3K/Akt pathway↑	viability↑, apoptosis↓, cisplatin cytotoxicity↓, glycolysis↑		[67]
Cholangiocarcinoma	CHD1L↑	hMLH1↓		Prognostic biomarker	[103]
	CHD1L↑	P53↓, cyclinD1↑, CDK2↑, E-cadherin↓, N-cadherin↑, Vimentin↑	EMT↑, G1/S transition↑, Cell proliferation↑	Poor prognosis	[20]

#### Prognostic biomarker and prognosis analysis of CHD1L

**Fig. 6:**
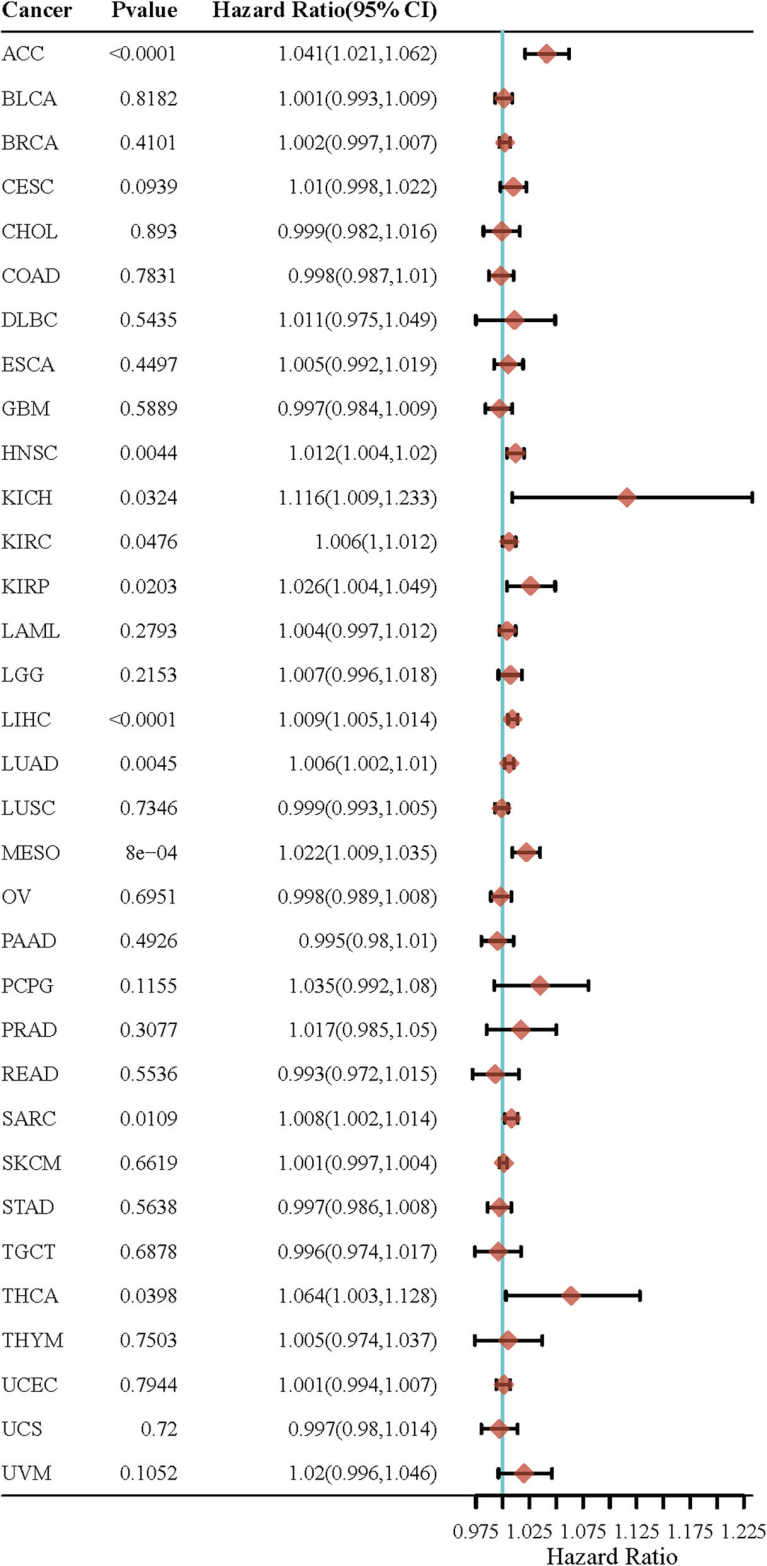
Prognostic analysis of CHD1L in TCGA database

CHD1L is considered a cancer or prognostic biomarker, because its overexpression is usually associated with poor prognosis, such as in HCC [21], gastric cancer [22], bladder cancer [25], breast cancer [26], NSCLC [12], cholangiocarcinoma [20], and nasopharyngeal carcinoma [27]. CHD1L expression is correlated with tumor size and stage in bladder cancer [25], nasopharyngeal carcinoma [27] and pancreatic cancer [65] in contrast to HCC [17] and ovarian cancer [23]. Further, the prognostic analysis of CHD1L was obtained from the TCGA database. The abnormal expression of CHD1L may be prognostic factor of ACC, HNSC, KICH, KIRC, KIRP, LIHC, LUAD, MESO, SARC and THCA (Fig. 6).

#### CHD1L and drug resistance

CHD1 as tumor suppressor gene, is currently rare in tumor resistance mechanisms. CHD1L as a potent anti-apoptotic and pro-proliferative factor, CHD1L overexpression is correlated with the increase of chemotherapy resistance in myeloma [6], NSCLC [66] and ESCC [67]. Bortezomib decreased the protein levels of CHD1L, but cell adhesion increased the expression levels of CHD1L, overexpression of CHD1L contributes to cell adhesion-mediated drug resistance (CAM-DR) in multiple myeloma cells [6]. Overexpression of CHD1L increased the transcription of c-Jun which targeted directly to the promoter of ABCB1 (initially isolated in drug-resistant Chinese hamster ovary cancer cells), and the upregulated ABCB1 phosphoried the NF-κB downstream Iκ-Bα and p65, therefore, CHD1L could induce cisplatin resistance via c-Jun-ABCB1-NF-κB axis in NSCLC [66]. Knockdown of CHD1L enhanced cisplatin cytotoxicity of ESCC cells by inhibition of glycolysis through inactivation of the PI3K/Akt pathway [67]. CHD1L results in cell malignant change and chemotherapy resistance through complex mechanisms in several solid tumors (Fig. 1b). Therefore, for patients with increased expression of CHD1L, effective inhibition of CHD1L may represent a promising therapeutic direction.

#### The pharmacological inhibition of CHD1L

Since CHD1L was discovered in 2008, it acts as an oncogene in a variety of tumors, but relatively few reported CHD1L as a druggable target and establishes a novel therapeutic strategy for the treatment of cancers. CHD1L possesses intrinsic poly (ADP)-ribose (PAR) chains via a C-terminal macro domain, which rapidly recruited to sites of DNA damage by PAR chains synthesized by PARP1/2 [11]. Therefore, PARP inhibitors may be applied to the target of CHD1L. Blessing et al. reveal CHD1L manipulation impacts the single-strand DNA break repair response and potentiates PARPi-induced cancer killing through PARP2 trapping [113]. PARP inhibitor Olaparib suppressed the DNA damage repair signaling and repressed the global pluripotent transcriptional network through CHD1L-mediated condensation of the chromatin structure in HCC [114]. Lead CHD1L inhibitors (Compounds 5–7) display potent antitumor activity by reversing TCF-driven EMT and induction of cleaved Ecadherin mediated extrinsic apoptosis in colorectal cancer [115]. The pharmacological inhibition of CHD1L might represent a promising therapeutic strategy for patients with decrease CHD1L expression. An overview of the CHD1L inhibitors is described in Table 4.

**Table 4:** Description of CHD1L-inhibitors identified so far

Compound	Chemical structure	Molecular Weight	Mechanism	Effect on CHD1L target genes	IC_50_	References
2-(4-Methoxyphenyl)-5-(methylsulfonyl)-4-(phenylsulfonyl)-1,3-oxazole		393.43	Inhibit that CHD1L binds the TCF complex, reverse TCF-driven EMT	TCF complex WNT response elements (WRE) (eg. c-Myc, vimentin, slug, LEF1, and N-cadherin)	3 μmol/L	[115]
N-(4-{[6-Methyl-2-(1-pyrrolidinyl)-4-pyrimidinyl]amino}phenyl)-2-(2-thienyl)acetamide		393.51			5.5 μmol/L	[115]
2-(4-{4-[(3-Chloro-4-methylphenyl)amino]-2-pteridinyl}-1-piperazinyl)ethanol		399.88			4 μmol/L	[115]
Olaparib		434.46	suppresses the DNA damage repair signaling, repress the key pluripotent transcriptional factors	DNA damage repair genes (eg. SSRP1,ERCC3,CHD1L,TP53BP1,TRIP13), the key pluripotent transcriptional factors (eg. SOX2, OCT4, c-MYC)	5–50 μM depending on Assays (HCC cells), 10–50 mg/kg (xenograft mouse model)	[113] [114]
Niraparib		492.59	suppresses the DNA damage repair signaling	DNA damage repair genes (eg. SSRP1,ERCC3,CHD1L,TP53BP1,TRIP13)	Not available (10 μM treated HCC cells)	[114]

## Conclusions

CHD1L is a multifunctional protein that plays important roles in normal or pathological conditions. However, how does CHD1L manage all those diverse functions? This may be related to its three main domains: the N-terminal domain of SNF2 family, HELICc and Macro domain. In normal cells, CHD1L mainly functions through the Macro domain. First, CHD1L recognizes PAR through the macro domain. PARP activates the chromatin-repositioning enzyme of CHD1L, which in turn activates PAR-dependent chromatin remodeling and promotes DNA repair. In addition, under pathological conditions, CHD1L is usually abnormally expressed in a variety of tumors. The mechanism of CHD1L involved in tumorigenesis may be the activation of certain genes (such as ARHGEF9, SPOCK1, TCTP) and/or suppression of other genes (such as p53, Nur77, hMLH1). The overexpression of CHD1L is also related to the clinical characteristics and prognosis of patients, so the expression level of CHD1L can be considered as a potential prognostic factor for cancer.

In conclusion, CHD1L recognizes several activities that play key roles in many biological processes. The significance of CHD1L in cellular and pathological effects have not been fully understood and remain to be explored. Therefore, this review suggests that CHD1L may be a novel biomarker for cancers and represents a fascinating target for molecular cancer therapy in the future.

## Data Availability

Not applicable.

## Abbreviations

CHD1L: Chromodomain helicase/ATPase DNA binding protein 1-like gene
ALC1: Amplified in liver cancer 1
SNF2: Sucrose non-fermenting 2
CGH: Comparative genomic hybridization
ARHGEF9: Rho guanine nucleotide exchange factor 9
SPOCK1: Sparc/osteonectin, cwcv, and kazal-like domains proteoglycan 1
TCTP: Translationally controlled tumor protein
ABCB1: ATP-Binding Cassette Sub-Family B Member 1
hMLH1: Human mutL homolog 1 gene
NTKL: N-terminal kinase like protein
TPM: Transcript Per Million
FPKM: Fragments Per Kilobase of exon per Million
ACC: Adrenocortical carcinoma
BLCA: Bladder Urothelial Carcinoma
BRCA: Breast invasive carcinoma
CESC: Cervical squamous cell carcinoma and endocervical adenocarcinoma
CHOL: Cholangiocarcinoma
COAD: Colon adenocarcinoma
DLBC: Lymphoid Neoplasm Diffuse Large B-cell Lymphoma
ESCA: Esophageal carcinoma
GBM: Glioblastoma multiforme
HNSC: Head and Neck squamous cell carcinoma
KICH: Kidney Chromophobe
KIRC: Kidney renal clear cell carcinoma
KIRP: Kidney renal papillary cell carcinoma
LAML: Acute Myeloid Leukemia
LGG: Brain Lower Grade Glioma
LIHC: Liver hepatocellular carcinoma
LUAD: Lung adenocarcinoma
LUSC: Lung squamous cell carcinoma
MESO: Mesothelioma
OV: Ovarian serous cystadenocarcinoma
PAAD: Pancreatic adenocarcinoma
PCPG: Pheochromocytoma and Paraganglioma
PRAD: Prostate adenocarcinoma
READ: Rectum adenocarcinoma
SARC: Sarcomav
SKCM: Skin Cutaneous Melanoma
STAD: Stomach adenocarcinoma
TGCT: Testicular Germ Cell Tumors
THCA: Thyroid carcinoma
THYM: Thymoma
UCEC: Uterine Corpus Endometrial Carcinoma
UCS: Uterine Carcinosarcoma
UVM: Uveal Melanoma
